# Biogenesis and Breakdown of Lipid Droplets in Pathological Conditions

**DOI:** 10.3389/fcell.2021.826248

**Published:** 2022-02-07

**Authors:** Claudio M. Fader Kaiser, Patricia S. Romano, M. Cristina Vanrell, Cristian A. Pocognoni, Julieta Jacob, Benjamín Caruso, Laura R. Delgui

**Affiliations:** ^1^ CONICET Dr. Mario H. Burgos Institute of Histology and Embryology (IHEM), Mendoza, Argentina; ^2^ Instituto de Investigaciones Biologicas y Tecnologicas, Facultad de Ciencias Exactas, Físicas y Naturales, Universidad Nacional de Cordoba, Cordoba, Argentina

**Keywords:** lipid droplet (LD), LD breakdown, LD biogenesis, protozoans, viral infection, cancer

## Abstract

Lipid droplets (LD) have long been considered as mere fat drops; however, LD have lately been revealed to be ubiquitous, dynamic and to be present in diverse organelles in which they have a wide range of key functions. Although incompletely understood, the biogenesis of eukaryotic LD initiates with the synthesis of neutral lipids (NL) by enzymes located in the endoplasmic reticulum (ER). The accumulation of NL leads to their segregation into nanometric nuclei which then grow into lenses between the ER leaflets as they are further filled with NL. The lipid composition and interfacial tensions of both ER and the lenses modulate their shape which, together with specific ER proteins, determine the proneness of LD to bud from the ER toward the cytoplasm. The most important function of LD is the buffering of energy. But far beyond this, LD are actively integrated into physiological processes, such as lipid metabolism, control of protein homeostasis, sequestration of toxic lipid metabolic intermediates, protection from stress, and proliferation of tumours. Besides, LD may serve as platforms for pathogen replication and defense. To accomplish these functions, from biogenesis to breakdown, eukaryotic LD have developed mechanisms to travel within the cytoplasm and to establish contact with other organelles. When nutrient deprivation occurs, LD undergo breakdown (lipolysis), which begins with the LD-associated members of the perilipins family PLIN2 and PLIN3 chaperone-mediated autophagy degradation (CMA), a specific type of autophagy that selectively degrades a subset of cytosolic proteins in lysosomes. Indeed, PLINs CMA degradation is a prerequisite for further true lipolysis, which occurs via cytosolic lipases or by lysosome luminal lipases when autophagosomes engulf portions of LD and target them to lysosomes. LD play a crucial role in several pathophysiological processes. Increased accumulation of LD in non-adipose cells is commonly observed in numerous infectious diseases caused by intracellular pathogens including viral, bacterial, and parasite infections, and is gradually recognized as a prominent characteristic in a variety of cancers. This review discusses current evidence related to the modulation of LD biogenesis and breakdown caused by intracellular pathogens and cancer.

## Introduction

Lipid droplets (LD) are multi-functional and highly connected organelles with a central role in cellular metabolism and homeostasis (Farese and Walther, 2009; Walther and Farese, 2012; Thiam et al., 2013b; Gao and Goodman, 2015; Welte and Gould, 2017; Olzmann and Carvalho, 2019; Beller et al., 2020). LD are ubiquitous in nature as regards cell and organism types. They present a wide range of sizes (from the nanometer order up to microns) and composition (Thiam and Beller, 2017), and have a simple and conserved particular structure consisting of a core of neutral lipids, primarily triacylglycerols (TAG), and sterolesters (SE) and are stabilized by a coating monolayer of phospholipids (PL) and specific proteins (Zhang and Liu, 2017). The mobilization of fat stores from LD is regulated by the metabolic and energy demands of the cell. When the energy demand increases, TAGs are broken down into fatty acids (FA) and the glycerol backbone, and the former enter cellular energy production pathways in the mitochondria. LD are also highly dynamic as their size, shape, and composition can vary under stress conditions, such as viral and microbial infections (Roingeard and Melo, 2017; Martins et al., 2018). Like other cell organelles, LD follow a biogenesis and degradation cycle, which contributes to LD homeostasis. In this review, we present an overview of the LD biogenesis and degradation processes as well as the mechanisms modulating their functioning in pathological conditions such as viral and protozoan infections and cancer.

### Biogenesis

The mechanism of formation and growth of LD has been considered a fundamental question in LD biology (Ohsaki et al., 2014), and their biogenesis and modulating factors have become a research special focus over the last years (Thiam and Forêt, 2016; Walther et al., 2017; Chorlay and Thiam, 2018; Chapman et al., 2019; Jackson, 2019; Zoni et al., 2019, Zoni et al., 2021; Nettebrock and Bohnert, 2020; Santinho et al., 2020). LD are closely associated to the endoplasmic reticulum (ER), where the enzymes catalyzing the last step of TAG and SE synthesis are located (Pol et al., 2014). Several LD biogenesis models have been described, which differ from each other in the way that the protuberance of non-polar molecules located inside the bilayer is detached from the ER membrane (Brasaemle and Wolins, 2012; Thiam et al., 2013b; Thiam and Forêt, 2016; Jackson, 2019). In some models, the detachment process is driven by proteins whereas in others, the PL demixing driven by the membrane curvature plays a fundamental role (Zanghellini et al., 2010). The first step of an initial protuberance formation has been studied in depth by molecular dynamics (MD) simulations, which demonstrated that triolein blisters form inside PL bilayers (Khandelia et al., 2010; Zoni et al., 2019, Zoni et al., 2020, Zoni et al., 2021), and by mathematical models describing their shape using mechanical constraints (i.e., parameters determining elastic free energies of the bilayer and the monolayers coating the LD) (Zanghellini et al., 2010; Deslandes et al., 2017; Choudhary et al., 2018). Some factors that have been considered for the formation of an initial droplet are the TAG lateral solubility in bilayers, the mechanical constraints of the bilayer for deformation and the wettability of the bilayer with TAG. Solubility is referred as the maximum amount of TAG molecules that can be arranged in parallel to PL molecules (with the glycerol backbone facing the water phase), which is known to be 3–4% at most (Hamilton, 1989; Li et al., 2003). At higher contents, TAG molecules, like other NL may segregate into the intrabilayer space (Hauß et al., 2002; Corvalán and Perillo, 2020; Zoni et al., 2021). However, the nucleation process is poorly understood (Santinho et al., 2020) and there is a lack of experimental approaches that can address this issue, as at this stage LD are below optical resolution. Although some studies (Hamilton, 1989; Li et al., 2003; Duelund et al., 2013) have clearly demonstrated the presence of a segregated TAG phase somehow incorporated inside multilamellar vesicles at a TAG content slightly above 3%, the methods employed in those studies did not allow to draw any conclusion about the distribution of the TAG domains. Recently, Caruso et al. have used phosphatidylcholine (PC)/TAG Langmuir films to describe the segregation of TAG as a function of the packing and the composition of the membrane. This approach supports the assumption that TAG molecules segregate into discrete TAG lenses, whose shape is determined by the interfacial tensions through the contact angle between the lenses and their surrounding membrane, that is, its wettability (Caruso et al., 2021). Other authors have previously shown that the subsequent steps in the biogenesis process (budding and protrusion) are determined by physics of wetting. Thiam et al. have proposed and examined how bilayers interfacial tensions (and PL composition) affect the contact angles of apolar droplets introduced into the intrabilayer space (Thiam and Forêt, 2016). It has been observed that a system with a lower wettability (lower bilayer interfacial tension) forms more marked protrusions and thus a higher tendency to budding (Ben M’barek et al., 2017). Furthermore, the authors described that the compositional asymmetry of the bilayer determines the direction of budding in experimental systems (Chorlay and Thiam, 2018). In the cell, budding is expected to occur towards the cytoplasm, with this process being determined by ER membrane asymmetry.

Considering this evidence, LD formation can be described as a four-step process, comprising nucleation, growth, budding and detachment. Besides, the modulation of this process by proteins is currently being studied considering this differentiation. The first two steps are strongly modulated by the ER membrane protein seipin, which is more abundant in tubules than in the rest of the ER. Furthermore, the initial stage of LD biogenesis is most frequent in ER tubules. Seipin is known to stabilize TAG clusters and promotes the recruitment of TAG into them, whereas mutated forms give rise to aberrant LD shape and number (Cartwright et al., 2015; Wang et al., 2016; Santinho et al., 2020). However, the mechanism underlying TAG recruitment and nascent LD stabilization remains unclear. Similarly, the fat storage-inducing transmembrane protein 2 (FIT2) drives the LD biogenesis by interacting with ER tubule-forming proteins and septins (Chen et al., 2021). The lipase phosphatase activity of FIT2 has been recently described suggesting a role in the maintenance of the phospholipid balance between the cytosol and the lumen facing the ER hemi-layers (Becuwe et al., 2020). Another protein that participates in the first stages of the synthesis of LD, ubiquitously distributed, is perilipin 3 (PLIN3), which protects TAG aggregates from lipolysis. This protein, present in the cytoplasm, accumulates in nascent LD immediately after TAG nucleation (Pol et al., 2014). This recruitment has been postulated to be controlled by a hydrophobic pocket of the protein (Ohsaki et al., 2006). Finally, not all LD detach from the ER (Mishra et al., 2016; Valm et al., 2017) and although the detachment mechanism remains to be elucidated, indirect evidences suggest a role of complex protein I, COPI; i.e., detachment is reversible, with the re-attachment requiring the COPI coatomer complex (Thiam et al., 2013a; Wilfling et al., 2014).

The mechanisms for protein targeting to LD are currently the focus of an expanding research field, although some underlying mechanisms have already been elucidated (Dhiman et al., 2020). Proteins that are targeted to LD are divided into class I and class II, according to where they come from, i.e., the ER bilayer surrounding the attached LD or the cytosol, respectively. Thus, seipin and PLIN3 are examples of each class of proteins playing a role in the initial instances of LD biogenesis. Targeting membrane proteins from ER bilayer to LD surface is a logistical challenge for cells (Dhiman et al., 2020). The biophysical properties of ER (bilayer) and LD (monolayer + inner TG) membranes would be a first selection barrier controlling what type of proteins will partition between both structures (Thiam et al., 2013b; Kory et al., 2016). In this sense, LD cannot accommodate proteins with transmembrane regions that span the thickness of a bilayer.

**FIGURE 1:**
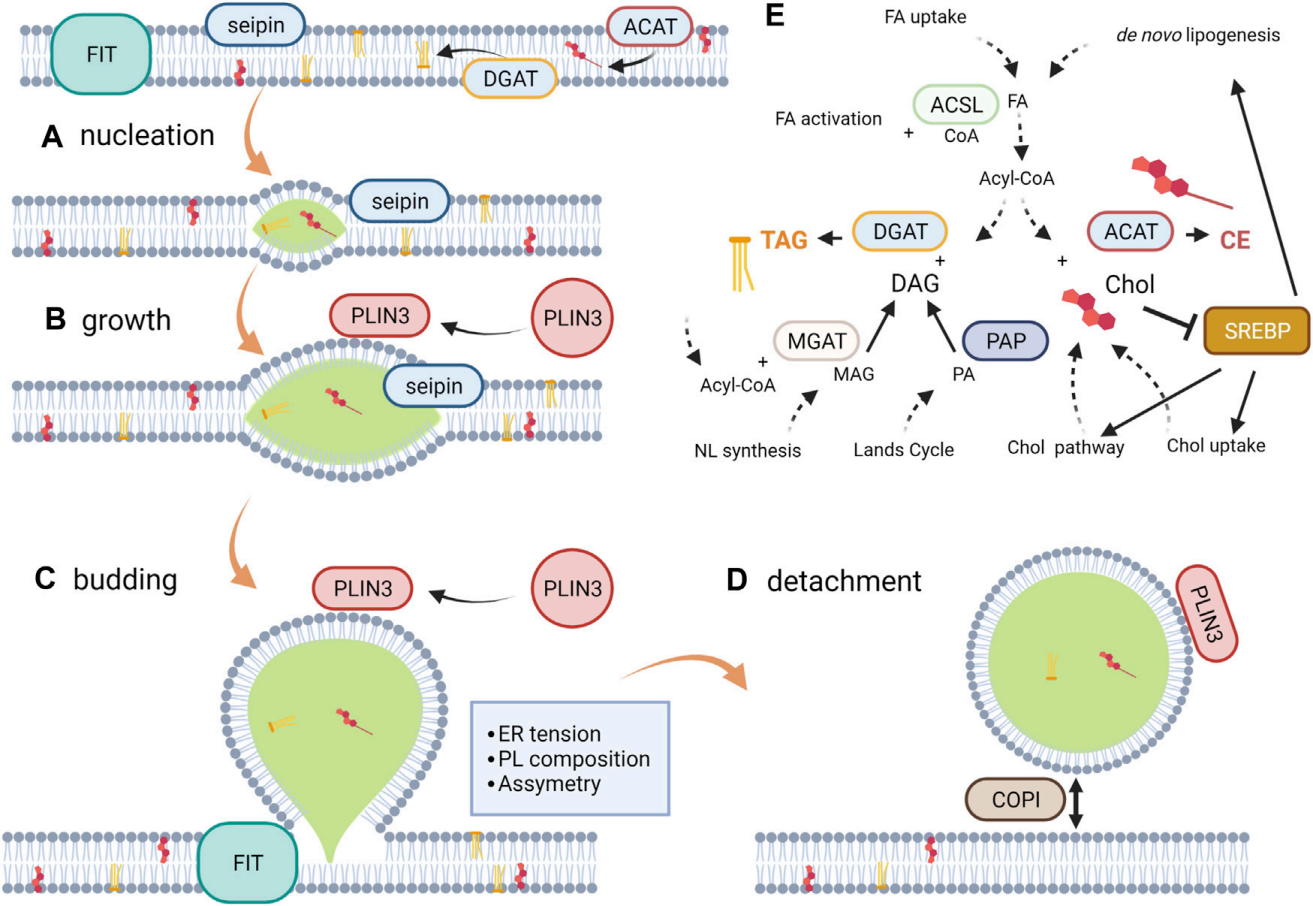
LD Biogenesis. From a biophysical perspective, LD biogenesis can be described as a four-step process. **(A)** Nucleation occurs when the ER bilayer saturates with NL, reaching a concentration in the bilayer to form lenses of the size in the order of nanometers. The PL composition and seipin among other proteins modulates this process through bilayer hydration, bending and curvature. **(B)** The individual lens growth takes place as newly synthesized NL are incorporated and by ripening and fusion between lenses; seipin prevents NL from shedding. **(C)** Budding is a spontaneous process (dewetting) driven by the interfacial tensions that come into play surrounding the droplet. The bilayer tension and the phospholipid asymmetry determine the sphericity and direction of budding, respectively. FIT has been recently proposed to be involved in the maintenance of the appropriate PL composition in each ER hemi-layer. **(D)** Although detachment is not to be expected for all LD, it is a reversible process. COPI has been proposed to be involved in the detachment/re-attachment process to the ER membrane. **(E)** Metabolic reactions more closely affecting NL synthesis and its modulating pathway SREBP. Note: the scheme represents a simplification of the metabolic vias according to the available evidence regarding the revised pathologies. For a more detailed description, see Pol et al. (2014).

The above considerations describe LD biogenesis mainly from a biophysical perspective. However, to determine the factors modulating this process, cell metabolism pathways must also be considered. For instance, the *de novo* lipogenesis is regulated by the intracellular concentrations of glucose and sterol via the carbohydrate responsive element binding protein (ChREBP) and the sterol regulatory element binding protein (SREBP). In the latter, a decrease in cholesterol levels and polyunsaturated FA (PUFA) facilitates the proteolysis of SREBP yielding transcription factors that activate the expression of components of the lipogenic pathway and cholesterol metabolism in a species-specific manner (Shimano and Sato, 2017). The *de novo* lipogenesis generates FA, which subsequently esterify the glycerol backbone in a series of steps shared by the phospholipid and neutral lipid synthetic pathways. The branching-off point between these pathways is the dephosphorylation of phosphatidic acid into diacylglycerol (DAG) by phosphatidate phosphatases (Zhang and Reue, 2017; Petan, 2020). Finally, other enzymes located in the ER membrane, namely diacylglycerol acyltransferase (DGAT) and acyl-CoA cholesterol transferases (ACAT) catalyze the last steps of TAG and cholesterol esters synthesis, respectively. The regulation of these pathways has been shown to be modulated both in infections and cancer. All these evidences have been incorporated into Figure 1.

### Breakdown

The catabolism of LD is regulated by the protein composition of the organelle surface and occurs by two mechanisms: lipolysis and lipophagy. As mentioned, LD have many different structural and functional proteins on its surface. In mammalian LD, the predominant proteins are the PLIN/adipose differentiation-related protein (ADRP)/tail-interacting protein of 47 kDa (TIP47) and their orthologs (grouped as the PAT family) (Miura et al., 2002; Bickel et al., 2009; Thiam et al., 2013b; Kory et al., 2016). In mammals, there are 5 different PLIN (PLIN1 to 5), among which PLIN1 and PLIN2 are exclusively associated with LD. The expression of PLIN1 is restricted to adipocytes and steroidogenic cells, while the expression of PLIN2 and PLIN3 are ubiquitously distributed (Sztalryd and Brasaemle, 2017). Among these proteins, PLIN regulate lipase access to the LD core; increased lipolysis in adipocytes was observed in their absence (Tansey et al., 2004). A general idea is that PLIN are needed to be somehow removed from the LD surface to “open a gate” for lipases to access the TAG (Brasaemle, 2013; Schweiger and Zechner, 2015).

Several LD proteins are degraded by the ubiquitin-proteasome system (UPS) under conditions of lipid starvation (e.g., cells cultured in the absence of FA supplementation), among which are PLIN1 (Xu et al., 2006) and PLIN2 (Xu et al., 2005; Takahashi et al., 2016). Lipolysis begins with the phosphorylation of PLIN1 by cAMP-dependent protein kinase A (PKA). Phosphorylated PLIN1 is then removed from the surface of LD for further proteasomal degradation, leading to the direct activation of LD-associated lipases: 1) patatin-like phospholipase domain containing 2 (PNPLA2/ATGL), which catalyzes the hydrolysis of TAG into DAG; 2) lipase E, hormone sensitive type (LIPE/HSL), which mediates the breakdown of DAG into MAG, and the hydrolysis of the ester bonds of other lipids such as SE, and 3) monoglyceride lipase (MGLL/MGL), which catalyzes the hydrolysis of MAG into glycerol and FA, which together with regulatory protein factors constitute the basis for this process (Zechner et al., 2012). The sequential action of the three lipases results in glycerol and FA generation. The products of lipolysis secreted from the adipose tissue are transported to other tissues and used for β-oxidation and ATP production. In non-adipose tissues, FA can enter the mitochondria directly for ATP production (D’Andrea, 2016).

Alternatively, PLIN2 and PLIN3, and more-recently described PLIN5, are substrates of lysosomal-degradation through a pathway named chaperone mediated autophagy (CMA) (Kaushik and Cuervo, 2015; Ma et al., 2020). CMA mediates the delivery of a subset of proteins exposing a pentapeptide motif (KFERQ or a related sequence) to the lysosome for proteolysis. In this process, heat shock cognate protein of 70 kDa (hsc70) recognizes, binds and delivers the protein to the lysosome-associated membrane protein 2A (LAMP-2A) within the lysosomal membrane, which forms a multimeric complex that translocates unfolded KFERQ-containing proteins into the lysosome lumen for degradation (Kaushik and Cuervo, 2018). CMA related-pentapeptides were identified in PLIN-2 (LDRLQ) and PLIN-3 (SLKVQ), and their degradation by CMA precedes ATGL-dependent lipolysis and lipophagy. Therefore, CMA is a crucial process in the degradation of LD (Schneider et al., 2014; Kaushik and Cuervo, 2015; Ma et al., 2020).

Macroautophagy, another autophagy-related pathway, constitutes an alternative route for the breaking-down of intracellular LDs and mobilization of lipid storage. In general, autophagy is one of the major degradation pathways that enables the cell to survive under stress conditions by recycling metabolic components with an especially relevant role in the degradation of hepatocellular LD (Singh et al., 2009; Van Zutphen et al., 2014). First described in mouse hepatocytes under starvation, a selective form of LD-targeting macroautophagy known as lipophagy is thought to involve the recognition of LD to promote the localized assembly and extension of a sequestering phagophore around the perimeter of the LD and their subsequent delivery to lysosomes for turnover (Singh et al., 2009; Singh and Cuervo, 2012; Schulze et al., 2017; Filali-Mouncef et al., 2021). How this phagophore is targeted to (and extended around) the LD surface to facilitate lipophagy remains unclear. However, it has been demonstrated that both ATGL and HSL, localized on the phospholipid monolayer limiting LD, contain several putative LC3-the major autophagosome marker-interacting regions (LIR) motifs. Co-immunoprecipitation experiments have revealed that these proteins interact with the microtubule associated protein 1 light chain 3 (MAP1LC3/LC3), and therefore could dock LD onto the cytoplasmic surface of phagophores (Martinez-Lopez et al., 2016). Once fully enclosed, the double-membrane vesicle named autophagosome undergoes fusion with the lysosome to form a degradative organelle known as an autolysosome. Lysosomal lipases within the autolysosome are then ultimately responsible for the acid hydrolysis of the LD-stored NL and subsequent release of free FA (Warner et al., 1981; Liu and Czaja, 2013; Gatica et al., 2018). Degradation products are then released back into the cytosol and can be reused by the cell for synthesis processes. The two pathways of lipolysis and lipophagy likely work in tandem as coordinated processes (Martinez-Lopez et al., 2016). Indeed, a different possible scenario is that lipolysis can act to rapidly reduce the size of large LD to diameters more appropriate in size for engulfment by lipophagic vesicles (Schott et al., 2019).

**FIGURE 2:**
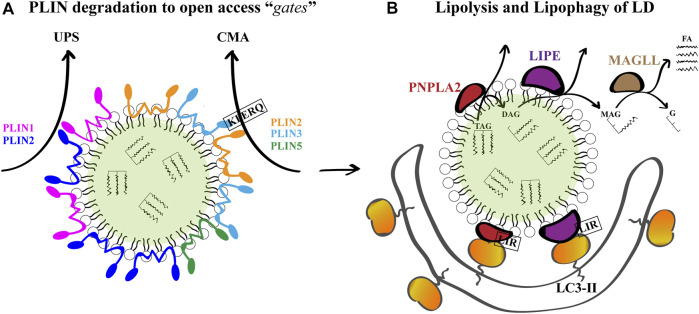
LD Breakdown. **(A)** Given that PLINs are gatekeepers of LD breakdown and further lipolysis, they must be degraded for breakdown to proceed. PLIN1 and PLIN2 are substrates of the ubiquitin-proteasome system (UPS), and PLIN2, PLIN3 and PLIN5 are substrates of chaperone mediated autophagy (CMA) under conditions of lipid demand. For PLIN2 and PLIN3, a KFERQ peptide has been demonstrated to mediate CMA. **(B)** The degradation of LD TAG and SE occurs by the sequential action of PNPLA2, LIPE and MGLL to produce glycerol (G) and free FA for further β-oxidation. Concomitantly, PNPLA2 and LIPE located on the LD surface can interact with LC3-II on phagophore membranes through their LC3 interacting regions (LIR) to promote lipophagy-mediated LD breakdown.

Among the vast repertoire of components that exquisitely regulate the autophagy pathway, Rab proteins, a family of small GTPases, act as important mediators of endosomal tracking events. Cycling between active GTP- and inactive GDP-bound states, Rab proteins regulate the vesicular tracking network within the cell (Stenmark, 2009). Numerous Rab proteins have been identified on LD, and changes in members of the Rab proteins family have deleterious effects on LD turnover in response to classical lipophagy-inducing stimuli (Kiss and Nilsson, 2014). The most conspicuous case is the presence of Rab7 on the LD surface. Rab7 is a well-characterized member involved in the control of late endocytic membrane trafficking (Vitelli et al., 1997), assisting the regulation of lysosome–autophagosome interaction (Gutierrez et al., 2004; Jäger et al., 2004). Moreover, Rab7 decorates the surface of LD and regulates macrolipophagy in mammalian cells (Schroeder et al., 2015). The Rab7 GTPase located on the surface of LD becomes activated upon nutrient deprivation, resulting in its increased activity for GTP over GDP. Such activated state promotes the recruitment of lysosomes near LD and their target degradation via lipophagy (Carmona-Gutierrez et al., 2015). All these evidences have been incorporated into Figure 2.

## Lipid Droplets and Protozoans

Protists are a heterogeneous group of ancient unicellular or pluricellular eukaryotes that can be divided into free living organisms and pathogenic parasites. The latter group encompasses organisms of Apicomplexa (*Toxoplasma gondii* and *Plasmodium falciparum*) and Kinetoplastida (*Trypanosoma cruzi*, *Trypanosoma brucei* and species of *Leishmania*) orders that infect humans causing the named Neglected Tropical Diseases (NTD), which are highly spread worldwide. These organisms possess the metabolic pathways for the production and breakdown of LD, like other higher eukaryotic cells. In the past, pathogen-derived LD were mostly considered as lipid deposits with low turnover rates (Murphy, 2012). In recent years, there has been an increasing interest in the study of these lipid-rich organelles present in pathogenic prokaryotes and lower eukaryotes. In this context, evidence begins to accumulate demonstrating that cytosolic LD of parasitic protozoans bear more dynamic roles in both, mammalian and non-mammalian stages of the parasite’s life cycle. Furthermore, they can interact with the LD of mammalian cells for their own benefit. In this section we present recent data demonstrating the role of LD during the biological cycle of protozoan pathogens (including the stages living inside and outside the host cells) and suggest the relevance of these compartments as targets of antiparasitic drugs.

### Lipid Inclusions of Reservosomes and Cytoplasmic LD are Key Components During *Trypanosoma cruzi* Differentiation and Host Cell Infection

*T. cruzi*, the etiological agent of Chagas disease, is one of the main causes of morbidity and mortality in Latin America. The life cycle of *T. cruzi* comprises three stages; epimastigotes and amastigotes are the replicative forms found in the intestine of the insect vector and in the cytoplasm of host cells, respectively; and the infective forms, metacyclic trypomastigotes and blood-stream trypomastigotes, which transmit the infection from the insect to mammals and *vice versa*. Two main compartments for lipid storage have been described in epimastigotes: the reservosome lipid inclusions and the cytoplasmic LD. The accumulation of cholesterol and SE within the reservosomes, the lysosome-like organelle of this parasite, is directly related to the host’s serum concentration of these metabolites (Pereira et al., 2011). Although *T. cruzi* cannot synthesize cholesterol, this compound is acquired through the uptake of low density lipoprotein (LDL) particles from the hematophagous insect diet (José Soares and de Souza, 1991). LDL are then transported in endocytic vesicles and delivered to reservosomes (Pereira et al., 2018). Inside the reservosome, LD are surrounded by a PL monolayer and display disk or rod-like shapes. Crystals of cholesterol were observed when cholesterol and SE masses reach a critical point, in a process that resembles the formation of foam cells in mammals (Varsano et al., 2018). In contrast, serum deprivation results in the consumption of the cholesterol storage of reservosomes (Pereira et al., 2015). Interestingly, it has been observed that as in higher eukaryotes, autophagy in *T. cruzi* is induced in response to nutrient starvation, leading to metacyclogenesis, the process of differentiation of epimastigotes to metacyclic trypomastigotes (Vanrell et al., 2017). This process is characterized by a dramatic reduction in the number of reservosomes, indicating that degradation of the reservosome content is a key step during differentiation, probably as an energy source (Cunha-e-Silva et al., 2002). Furthermore, it has been demonstrated that the induction of autophagy during metacyclogenesis increases the proteolytic activity of reservosomes, mainly due to cruzipain, which is the major cysteine-protease of *T. cruzi* and also an important virulence and immune evasion factor (Losinno et al., 2021). Therefore, it is reasonable to hypothesize that the degradation of LD in reservosomes could also be important during metacyclogenesis as an energy, cholesterol and other precursors source to generate the membranes of metacyclic trypomastigotes. *T. cruzi* also presents many uncharacterized LD distributed throughout its cytoplasm. During lipid starvation, cholesterol of reservosomes is mobilized and inserted into the membranes to maintain parasite proliferation, whereas under normal conditions, esterification reactions predominate, most likely to remove the excess of free cholesterol, leading to the formation of cytoplasmic LD (Pereira et al., 2015). The finding of enzymes involved in lipid metabolism, methyltransferases, reductases, lipases, and proteins like Rab18, and the ATP-binding cassette transporter 1, associated with the cholesterol efflux in humans, in reservosomes supports this hypothesis (Torres et al., 2004; Sant’Anna et al., 2009). The activity of an ACAT sensitive to avasimibe was also found in *T. cruzi* (Pereira et al., 2015). ACAT most likely function is to remove the excess of free cholesterol of reservosomes, leading to the formation of LD in *T. cruzi*. Aspartyl-like peptidases and cruzipain are also involved in cholesterol mobilization as shown by the accumulation of rod-shaped and droplet-shaped LD in reservosomes when the parasites are incubated with pepstatin-A, a typical aspartyl-peptidase inhibitor (Sangenito et al., 2021). Interestingly, this effect is imitated by lopinavir and nelfinavir, two Human Immunodeficiency Virus peptidase inhibitors with a high impact in *T. cruzi* viability (Sangenito et al., 2021). Although the connection between these peptidases and lipid accumulation is poorly understood, some authors have postulated that aspartyl-like peptidases present in the reservosomes could be directly and/or indirectly linked to the process of cholesterol mobilization by the endocytic pathway in the protozoan (Lechuga et al., 2020). *T. cruzi* trypomastigotes and amastigotes also have LD. In trypomastigotes, LD increase after both host interaction and exogenous arachidonic acid (AA) stimulation. Notably, AA-stimulated trypomastigotes release high amounts of prostaglandin E2 (PGE2) and show PGE2 synthase expression (Toledo et al., 2016). Although PGE2 actions are mainly proinflammatory, different authors propose an immunomodulatory effect that could contribute to the immunosuppression observed during *T. cruzi* infection, thus risking the survival of the parasite within its host. On the other hand, it is known that the *T. cruzi* trypomastigotes release extracellular vesicles with different functions, favoring the biosynthesis of LD and PGE2 in the host cell and reducing the production of inflammatory cytokines and trypanocide molecules such as nitric oxide, thus making the environment more favorable for the infection (Lovo-Martins et al., 2018). There is evidence showing that the infection of macrophages with trypomastigotes causes an increase in LD biogenesis in a Toll-like receptor (TLR) 2-dependent mechanism, since this process is not observed in bone marrow macrophages derived from C57BL/6 TLR2 knock out mice (TLR2−/−). D’Avila et al. (2011) have demonstrated that Toll-like receptor 4 does not participate in this process. It is known that TLR2, TLR3, TLR4 and TLR7 agonists increase the levels of proteins that are crucial for LD biogenesis (PLIN2 or DGAT2); however, in *T. cruzi* infected macrophages this effect is elicited only by TLR2, which, together with TLR9, plays a role in the immune recognition of this parasite (Tarleton, 2007). Interestingly, in contrast to LD from other host cells, LD from macrophages contain AA which is used to produce eicosanoids that are used in *T. cruzi* metabolism (den Brok et al., 2018). Thus, the treatment of macrophages with C75 (a FA synthase inhibitor) inhibits LD biogenesis and also induces a downregulation of eicosanoid production and replication of the parasite (de Almeida et al., 2018).

### *Trypanosoma brucei* Replication Requires the Rapid Turnover of Parasite LD

*T. brucei* is the causative agent of sleeping sickness or African trypanosomiasis, a disease characterized by behavioral abnormalities such as somnolence during daytime. Unlike all other pathogenic trypanosomatids which have an intracellular life-stage, *T. brucei* infection takes place in the bloodstream of mammalian hosts. After a blood feed of the tse-tse vector fly, metacyclic trypomastigotes reach the bloodstream of mammals and differentiate into long-slender trypomastigotes with high replicative capacity, followed by a second differentiation step into non-replicative short-stumpy forms when parasite density increases. The cycle is completed when blood trypomastigotes ingested by flies transform into procyclic trypomastigotes which migrate from the midgut to the salivary gland of the insect where they undergo differentiation to infective metacyclic trypomastigotes. The biogenesis of LD in *T. brucei* depends on the activity of a novel LD Kinase (LDK). The association of LDK with LD is apparently mediated by its hydrophobic domain that allows the insertion into the membrane monolayer of the organelles. The loss of this enzyme dramatically decreases the abundance of LD and affects the growth of parasites in delipidated serum (Flaspohler et al., 2010). It has been postulated that the function of FA storage in LD is to enable the adaptation of procyclic trypomastigotes to nutritional challenges during the development and migration inside the tse-tse fly. Under physiological conditions, FA (oleate) are taken from the medium and incorporated to TAG in the LD of procyclic trypomastigotes (Allmann et al., 2014). Plasma membrane PL provide the precursor DAG for esterification. Nejad et al. have shown that the *T. brucei* lipin homolog TbLpn is essential for parasite survival in culture. Lipins are a family of phosphatidic acid phosphatases that catalyze the dephosphorylation of PA to DAG. It has been demonstrated that the inducible downregulation of TbLpn decreases the number of LD and reduces TAG steady-state levels (Dawoody Nejad et al., 2018). These authors hypothesized that the rapid lipid turnover observed in their experiments could be required for the synthesis or remodeling of membrane lipids during cell proliferation, or for energy supply under limited nutrient availability. Apart from TAG, *T. brucei* also produces SE. Although a putative gene for the ACAT enzyme has not been found in *T. brucei*, its presence was evidenced by the production of SE from host LDL particles and subsequent accumulation in parasite LD, as it occurs in *T. cruzi* (Coppens et al., 1995).

### LD Biogenesis Increases During *Leishmania* Metacyclogenesis and Contributes to the Infection Process

Leishmaniasis is a disease caused by more than twenty species of the *Leishmania* genus. Distributed worldwide, this disease can be found in three clinical forms: visceral leishmaniasis, the most severe illness, is highly endemic in the Indian subcontinent and East Africa; cutaneous leishmaniasis, which occurs in the Americas, the Mediterranean basin, the Middle East and Central Asia; and mucocutaneous leishmaniasis, mainly occurring in Brazil, Bolivia, Ethiopia and Peru. The infection is transmitted to mammals by metacyclic promastigotes present in the proboscis of sandflies, which are the vectors of the disease. Metacyclic promastigotes are phagocytized by macrophages and differentiate into amastigotes that replicate in phagolysosomal compartments, releasing the parasite after macrophage lysis, a process that causes tissue damage (Pace, 2014). After a blood feed, amastigotes are taken by the vector and differentiate in procyclic promastigotes which proliferate in the insect gut and then migrate to the proboscis where they differentiate into metacyclic promastigotes. It has been demonstrated that LD numbers increase during *Leishmania* metacyclogenesis, i.e., the transformation of procyclic to metacyclic promastigotes. LD from metacyclic forms contain PGF2α synthase (PGFS) and release PGF2α in higher quantities than in other procyclic or amastigote forms, suggesting a role of PGF2α in parasite virulence (Araújo-Santos et al., 2014). On the other hand, the cholesterol supply is assured by the uptake and retention of LDL particles in *L. amazonensis* lipid membrane microdomains. BODIPY-labeled LDL is distributed in large compartments along the parasite body. These compartments also contain SE, suggesting the presence of an ACAT enzyme similar to *T. cruzi* and *T. brucei* (De Cicco et al., 2012). Furthermore, when metacyclic promastigotes of *Leishmania major* infect the bone marrow-derived macrophages (BMM), an increase in the LD biogenesis in these host cells is observed as a function of time. This increase is due to an induction of the expression of genes involved in cholesterol uptake and *de novo* synthesis of TAG in BMM infected with *L. major*. By a microarray assay, the authors demonstrated the transcriptional activation of several genes of BMM such as *DGAT2*. This host cell response occurs regardless of the viability of the parasites, as it occurs in either living or dead parasites and even in uninfected neighboring cells, although at a lesser extent than in infected cells, which would indicate that the phagocytosis of this parasite further increases the biogenesis of LD (Rabhi et al., 2016).

### *Toxoplasma gondii* Incorporates Host LD in its Parasitophorous Vacuole Favoring Parasite Replication

*T. gondii* is the etiological agent of toxoplasmosis, a severe disease in individuals with an impaired immune system, mainly producing neurological complications, and in the fetus that becomes infected by vertical transmission during pregnancy. In the latter case, *T. gondii* can cause severe neurological, ocular and cardiac disorders (Paquet et al., 2013; Laboudi, 2017; Fard et al., 2020). *T. gondii* is an obligate intracellular parasite with a complex life cycle involving one feline host, where the parasite sexual phase occurs, and intermediate hosts including humans. In humans, parasite transmission occurs through the ingestion of either raw or undercooked meat containing tissue cysts with bradyzoites; the ingestion of water and food contaminated with feline feces containing oocysts with sporozoites; and through transplacental transmission of tachyzoites during pregnancy. After ingestion, bradyzoites and sporozoites invade intestinal epithelial cells and differentiate into the fast-replicating tachyzoites inside a parasitophorous vacuole. In the acute infection, tachyzoites exit the cells, reach the bloodstream, and disseminate throughout the body. In healthy adults, cysts containing slow-replicating bradyzoites are located in the eyes, brain and muscles in the chronic phase of the disease, while in immunocompromised patients, the infection becomes severe and even fatal, as mentioned above (Attias et al., 2020). A DGAT1-like enzyme, required for the synthesis of NL, was characterized in *T. gondii* (TgDGAT1). This enzyme, localized in the parasite cortical and perinuclear ER, synthesizes TAG and generates cytosolic LD (Quittnat et al., 2004). As in other pathogenic protozoans, cholesterol is incorporated in *T. gondii* from the host environment. In this sense, two ACAT-related enzymes were identified and characterized in this parasite, TgACAT1α and TgACAT1β (also named TgACAT1 and TgACAT2). These enzymes localize to the ER and participate in the SE and LD synthesis (Nishikawa et al., 2005; Lige et al., 2013). Genetic ablation of each individual ACAT results in impairment of parasite growth, whereas dual ablation is not tolerated by *T. gondii*, thus highlighting the key role of cholesterol storages and LD in this organism and the possibility to consider this system as a target for new antitoxoplasmosis drugs (Lige et al., 2013). As for host cell stages, an increase was observed in the synthesis and accumulation of TAG when skeletal muscle cells (SkMC) are infected with *T. gondii* tachyzoites (Hu et al., 2017; Nolan et al., 2017), which is related to an increase in the number and size of LD in the host cells (Gomes et al., 2014; Nolan et al., 2017). There is evidence indicating that effectors synthesized by the parasites and exported from the PV to the host cell cytosol are responsible for the increase in the number of LD, since the knockout of the MYR1 protein (involved in the export of other PV effectors to the cytosol) is necessary for the accumulation of LD induced by this parasite. Other authors have demonstrated a role of the c-Jun kinase and the mammalian target of rapamycin (mTOR) signaling pathways in the modulation of parasite-induced generation of LD, which were supposed to provide nutrients to the parasite, since the pharmacological inhibition of these pathways did not produce an accumulation of LD during infection (Hu et al., 2017). Accordingly, it has been shown that the replication of *T. gondii* decreases when host cell LD are scarce, for example when the enzyme DGAT is inhibited (Nolan et al., 2017). It has also been observed that these LD were in contact with the ER and with the PV containing the parasite (Gomes et al., 2014). Other authors have described the presence of host LD within the PV and even in the parasite’s cytoplasm, suggesting that *T. gondii* can access and incorporate host lipids to its own membranes and LD (Nolan et al., 2017). Interestingly, although the increase in LD numbers is beneficial for the parasite as a nutrient source, LD serve as substrate for the production of PGE2, which is a crucial metabolite for the synthesis of interleukin-12 and interferons that participate in the repair and homeostasis of SkMC after the injury caused by the parasite, and contributing to the establishment of the chronic phase of infection (Gomes et al., 2014). Other parasites of the same family (*Sarcoystidae*) also induce the formation of host LD, like *Neospora caninum*, an Apicomplexa parasite of livestock and domestic animals, which is known to increase the levels of TAG and LD in human fibroblasts after infection (Hu et al., 2017).

### Biogenesis and Breakdown of Host LD are Required During the Intraerythrocytic Development of *Plasmodium*

*Plasmodium* species are the causative agents of malaria, the illness with the highest morbidity rates among human parasitic diseases. Currently, five identified species of *Plasmodium* infect humans, with *P. falciparum* being the most lethal. *Anopheles* spp. mosquitoes are the host that transmit the infection to humans (intermediary hosts) through the inoculation of sporozoites which migrate and develop in the liver. After invasion of hepatocytes, infective elongated sporozoites start the asexual multiplication and form the squizont. Sporozoites inside the squizont then form daughter cells called merozoites which, after maturation, are released from hepatocytes enclosed in a membrane (merosome). After merosome lysis, free merozoites invade red blood cells and transform into round proliferative trophozoites that mature into an erythrocytic schizont, which in turn rupture and release merozoites (Maier et al., 2019). A proportion of parasites differentiate into gametocytes (sexual forms) which are taken up by a mosquito when it feeds on human blood. Gametocytes undergo sexual reproduction in the midgut of the mosquito and develop into sporozoites, which migrate to the salivary glands to start a new cycle. The metabolism and trafficking of TAG and host LD in infected erythrocytes varies in a specific way during the intraerythrocytic cycle of this parasite. Increased DGAT activity and accumulation of TAG was observed during the development of *Plasmodium* from the trophozoite to squizont form, whereas TAG degradation was induced during the fragmentation of the squizont, with FA being released to the medium together with merozoites (Palacpac et al., 2004). In line with these observations, LD within red blood cells increase in size and number during the intraerythrocytic development, reaching a maximum number in the segmented schizonts stage. Interestingly, in the intraerythrocytic stage of *P. falciparum*, a population of Nile Red-positive particles was observed within the digestive vacuole of the parasite. These particles are composed of NL. These NL-rich particles, which may have originated from the digestion of previously internalized PL by the food vacuole (Jackson et al., 2004), have a key role in heme detoxification through the formation of the insoluble malaria pigment hemozoin (Hoang et al., 2010). These findings support the hypothesis that the storage and degradation of TAG are important processes during merozoite maturation and that NL present in the parasite food vacuole prevent heme toxicity. In contrast to the described role of LD in the erythrocytic cycle of *Plasmodium*, there is no evidence supporting the existence of a metabolism and trafficking of LD in the intrahepatic infection that occurs before intraerythrocytic phase.

**FIGURE 3:**
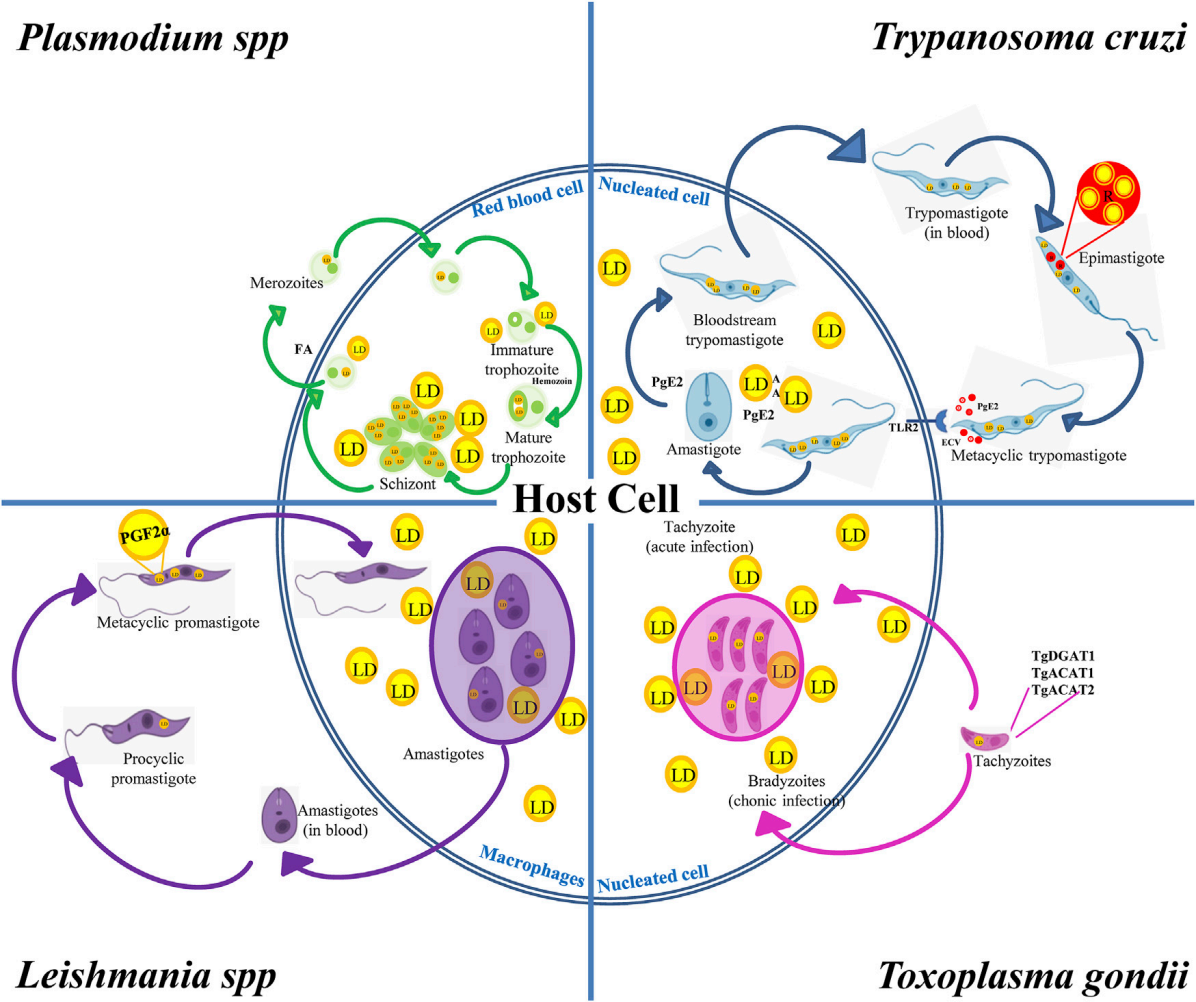
LD in the biological cycles of protozoan parasites. *Trypanosoma cruzi* displays reservosomes (R) in epimastigotes and cytoplasmic LD in metacyclic trypomastigotes (MT) that mainly store cholesterol and SE, respectively. While degradation of R occurs during metacyclogenesis, the contact with the host cell induces the production of LD and PGE2 in MT and in the host cell favoring *T. cruzi* infection and replication. *Toxoplasma gondii* expresses TgDGAT1, TgACAT1 and TgACAT2, which are the enzymes responsible for the LD synthesis in the parasite. An increased number of host LD was observed in cells containing vacuoles with tachyzoites, which is the characteristic parasite stage of the acute infection. These LD are also observed in the vacuole lumen and inside the parasite cytoplasm, thus evidencing the transport of host lipids to the parasite. *Leishmania* spp. increases the LD number during the evolution from procyclic to metacyclic promastigotes, which also contain PGF2α, suggesting a role of this eicosanoid as a parasite virulence factor. Host’s LD are also increased in macrophages infected with *Leishmania*. Like *T. gondii*, these host LD have been observed inside the *Leishmania* vacuole and even in the parasite’s cytoplasm. *Plasmodium* was found to store NL in the food vacuole. NL are important to prevent heme toxicity by production of the malaria pigment hemozoin. Infected red-blood cells increase the number and size of LD when they evolve from the trophozoite to squizont form, whereas LD breakdown characterizes merozoite maturation and release together with FA.

In summary, pathogenic protozoans can produce and degrade their own LD and interact with the host’s LD throughout their biological cycle. While parasite LD favor stage differentiation and infection stages, host LD are nutritional sources during their intracellular replication. Like in mammalian cells, parasite LD are distributed in the cell cytoplasm, although in some cases they are present inside other organelles such as lipid inclusions inside reservosomes of *T. cruzi* or NL particles in the *Plasmodium* food vacuole. LD biogenesis is induced after the acquisition of lipids (e.g., cholesterol and FA) from the external environment, blood of mammalian hosts or insect vectors mainly by endocytosis. DGAT and ACAT present in protozoans then lead to DAG and cholesterol esterification for final storage in LD. A few studies have addressed the catabolism of LD in these parasites; however, this process is known to be induced at specific developmental stages during the differentiation from a parasitic form to another, suggesting a role of LD in the energy supply required for the process. Interestingly, many of the enzymes involved in LD metabolism in protozoan parasites are vital for the organism, for they are unique, unlike their mammalian counterparts (Lige et al., 2013; Dawoody Nejad et al., 2018). Therefore, specific inhibitors of these enzymes could be interesting targets of drugs to interrupt the biological cycle of pathogenic protozoa, mainly in the mammalian stages. On the other hand, *T. cruzi*, *Leishmania* spp., *T. gondii* and *Plasmodium* spp., have intracellular stages that generate changes in the number and size of the LD of the host cell. It has been shown that LD increase in number and size when these intracellular parasites interact with the host cell. There is increasing evidence that protozoan parasites may target these host-derived LD to obtain nutrients for growth. However, host cells use the lipids stored in LD to produce inflammatory mediators against these parasites (Melo et al., 2003; D’Avila et al., 2012; Gomes et al., 2014; de Almeida et al., 2018). Due to the modulation of LD number by intracellular forms of protozoans that can determine the success or failure of the infection, the parasite/host LD interplay might be an attractive target to exploit in the future. All these evidences have been incorporated into Figure 3.

## *Flaviviridae* and Lipid Droplets

*Flaviviridae* is a large group of enveloped viruses, with a positive sense single strand RNA genome. The *Flaviviridae* family includes several viruses that cause high clinical impact diseases in humans: hepatitis C virus (HCV) of the *Hepacivirus* genus, yellow fever virus (YFV), West Nile virus (WNV), dengue virus (DENV) and Zika virus (ZIKV) belonging to the *Flavivirus* genus (Wu et al., 2015). Several studies have shown that members of the *Flaviviridae* virus family hijack the LD machinery for the replication and production of new mature viral particles. So far, the *Hepacivirus* HCV, and the *Flavivirus* DENV and ZIKV, are the most studied viruses as regard as LD usurpation (Filipe and McLauchlan, 2015; Sun et al., 2017; Cloherty et al., 2020).

*Hepacivirus* and *Flavivirus* genera share almost the same number and structural distribution of their proteins: a capsid protein (*Core* for HCV and *C* for *Flavivirus*), two envelope proteins (E1 and E2 for HCV, and prM and E for *Flavivirus*) and several non-structural proteins (NS) (Neufeldt et al., 2018). On the other hand, their replication cycle are quite similar: they first interact and enter the target cell by receptor mediated endocytosis, and after fusion with the lysosomes and acidification they uncoat and release their genome for translation of the viral polyprotein. At this point, the LD and ER play a key role as a scaffold for the newly synthetized virus assembly. Afterwards, the virion moves into the ER-Golgi lumen for proper assembly and maturation, and the final mature viruses are released through the secretory pathway to the extracellular space to start a new infective cycle (Zeisel et al., 2013; Guzman and Harris, 2015; Musso and Gubler, 2016).

### HCV and DENV/ZIKV Hijack the SREBP Pathway to Accomplish Viral Replication

The SREBP pathway is a key regulator of cholesterol/lipid levels, and therefore a key pathway for LD homeostasis (Eberle et al., 2004; Moon, 2017). Several studies have shown that some members of the *Flaviviridae* family hijack SREBP for their own benefit. Particularly, it has been shown that HCV and DENV/ZIKV trigger SREBP activation in order to fulfil the extra-membrane requirements during the cellular infection, replication and production of new virions (Randall, 2018; Meng et al., 2019; Yuan et al., 2019; Cloherty et al., 2020; Raini et al., 2021).

HCV is the most studied flavivirus hijacking the SREBP pathway. In this regard, HCV usurps and stimulates SREBP by disruption of the lipid homeostasis, generating a membranous web and activating the transcription of SREBP target genes for the final release of lipid-coated lipoviroparticles (LVPs) (Waris et al., 2007). In fact, clinical studies have shown that patients with chronic HCV infection resulted in a reduction of their circulating lipid levels (lower LDL and total cholesterol levels) as compared to patients developing a sustained virologic response (SVR) (patients with non-detectable HCV RNA after the completion of the antiviral therapy). This phenomenon might be explained by the extra lipid consumption that the HCV infection generates, re-routing the circulating lipids for the formation of LVPs (Corey et al., 2009). LVPs are hybrid particles composed of viral components (E1, E2, core protein and HCV RNA) and apolipoproteins (ApoE, ApoB, ApoCI, ApoCII and ApoCIII) (Scholtes et al., 2012). The presence of these apolipoproteins on the HCV surface has been proposed as a viral strategy to hijack neutralizing antibodies (Vercauteren et al., 2014). Particularly, the HCV core protein, the non-structural proteins NS4B, NS5A and the 3′ untranslated region (UTR) increase the activation of both SREBP-1 and SREBP-2, stimulating the synthesis of cholesterol and membrane lipids. In addition, it has been reported that HCV infection induces SREBPs cleavage and phosphorylation. In this regard, HCV core and NS4B proteins can induce the proteolytic cleavage of SREBP and oxidative stress by activating the phosphatidylinositol 3-kinase (PI3-K)-protein kinase B (PKB, also known as AKT) pathway, increasing phosphorylation and transactivation of SREBPs (Waris et al., 2007).

As mentioned, AMPK is an important sensor for cellular energy levels and has been demonstrated to be involved in lipid metabolism regulation. In a recent publication, HCV NS5A was shown to inhibit AMPK phosphorylation *in vivo* and *in vitro*, resulting in an increase of SREBP-1c expression levels, acetyl-coenzyme A carboxylase 1 (ACC1) and FA synthase (FASN) via the AMPK/SREBP-1c pathway, generating at the same time, higher numbers of LD (Meng et al., 2019).

The ATP-dependent RNA helicase DEAD-box helicase 3 X (DDX3X) is involved in cellular processes that are different from those involved in innate immunity. DDX3X is required for the replication of many viruses including HCV (He et al., 2021; Winnard et al., 2021). DDX3X has been shown to colocalize with LD, HCV core and NS proteins. However, DDX3X specifically recognizes the HCV 3′ untranslated region (UTR) in the cytosol and after cross-activation of IκB kinase-α (IKKα), it translocates to the nucleus and activates SREBPs. Thus, the LD biogenesis and HCV viral assembly are stimulated (Li et al., 2013; Pene et al., 2015). In summary, these findings highlight that LD are necessary and that their synthesis is stimulated during the HCV infection lifecycle.

The role of the SREBP pathway in *Flaviviridae* infection has been determined in several studies, in which different inhibitors of this pathway were used. In the case of the HCV infection, the SK1-1/S1P pathway was blocked with PF-429242 (a small, site-directed, competitive inhibitor of SKI-1/S1P). The treatment with PF-429242 led to a reduction of LD formation and an impairment in the early steps of HCV lifecycle was shown at all the inhibitor concentrations employed (Olmstead et al., 2012; Blanchet et al., 2015). PF-429242 showed a similar inhibitory profile for DENV and ZIKV, causing a marked reduction in the number of LD and LD-positive areas and a significant reduction in the viral titer in all the treated cell lines (Hyrina et al., 2017; Raini et al., 2021). In accordance, the AM580 (a retinoic acid receptor α selective agonist) binds SREBP1/2, showing an antiviral effect against a wide range of viruses including ZIKV. AM580 would then block the interaction between SREBP and the sterol regulatory elements (SREs) of the genes involved in lipid biosynthesis. Therefore, gene transcription is inhibited, with the consequent inhibition of ZIKV replication (Yuan et al., 2019). In a follow-up study, a link between the SREBP pathway and the antiviral protein STING (stimulator of interferon (IFN) genes) was proposed in DENV infection (Liu et al., 2017). In that work, the protein SCAP (SREBP member) was shown to bind and block the DENV protease NS2B3, thus inhibiting the cleavage of STING and impairing DENV infection. Interestingly, the authors also found that the ectopic expression of SCAP inhibited DENV infection, whereas the knockdown of this protein did not cause any effect on DENV lifecycle (Liu et al., 2017).

In conclusion, these facts highlight the importance of SREBP upregulation in HCV and DENV/ZIKV replication. During these infections an extramembrane and LD requirement appears to be crucial for the viral lifecycle. However, a better understanding of this pathway is necessary to provide a more detailed description of the molecular interactions between LD and some *Flaviviridae* members.

### Nuclear Lipid Droplets and HCV or the DENV/ZIKV Interaction May Contribute to the Viral Hijacking Process

Although initially LD were proposed to localize only to the cytoplasm of eukaryotic cells, later reports confirmed that these structures can also be found in the nucleus (nuclear lipid droplets, nLD). Nuclear LD are dynamic organelles storing neutral lipids originated from the inner nuclear membrane (INM). Nuclear LD have been proposed to act as an endonuclear buffer system, either providing or accepting lipids and proteins in different signaling pathways (Lagrutta et al., 2021). However, they can also be found attached to the INM in some processes by the transmembrane protein seipin (Romanauska and Kohler, 2018). Because nLDs have been described recently, very little is known about their role in the *Flaviviridae* replication cycle. Several studies indicate that HCV and DENV/ZIKV capsid and NS proteins may localize to the nucleus in infected cells (Majumder et al., 2001; Falcon et al., 2005; Netsawang et al., 2010; Garcia et al., 2020). It has been suggested that these viral proteins might interact with nLD, thus prolonging the interaction time and permanency with different host nuclear proteins. In turn, this phenomenon may allow an extended viral hijacking time of cellular metabolic pathways (Cloherty et al., 2020). In fact, recent publications suggest that the co-localization of nLD with *Flaviviridae* core proteins and the non-structural proteins NS5A (HCV) or NS5 (DENV/ZIKV) may represent a novel way to either induce (DENV/ZIKV) or inhibit (HCV) host cell apoptosis, as well as to create a link with viral release, carcinogenesis induction or impairment of the cellular interferon response (Ng et al., 2003; Herzer et al., 2005, Herzer et al., 2012; Heaton and Randall, 2010; Zhang and Wang, 2012; Liang et al., 2016; Wu et al., 2021).

Summarizing, the targeting of some *Flaviviridae* components to nLD may represent a novel understudied viral hijacking mechanism, in which apoptosis might also be involved. However, further studies assessing the interaction of nLD with *Flaviviridae* proteins may contribute to the understanding of the mechanisms by which viral infections progress to apoptosis, cancer or even steatosis in different cell types.

### Role of Lipophagy During HCV and DENV/ZIKV Infection

According to many authors, lipophagy refers to the catabolic process by which internal cell lipids stored in LD can be directed to lysosomes for final degradation by autophagy to release FA, and subsequently be processed via β-oxidation to provide energy for viral infection and replication processes (Singh et al., 2009). In fact, it has been reported that some of the *Flaviviridae* members may alter the autophagy degradative process to their advantage (Liang et al., 2016; Chan and Ou, 2017; Zhang et al., 2018). For instance, HCV, hijacks autophagy in order to promote the translation of its RNA and allow viral replication (Dreux et al., 2009). In addition, the proteins Beclin1and ATG7, involved in the autophagosome biogenesis, have been shown to be crucial for the release of mature HCV particles in Huh-7 infected cells, since the knocking down of Beclin1 or ATG7 causes a marked accumulation of HCV viral particles inside infected cells (Shrivastava et al., 2016). In a follow-up study, it was demonstrated that HCV infection upregulated autophagy at early steps of the infection cycle (Chan and Ou, 2017).

However, there are few studies showing contradictory results regarding how HCV may modulate lipophagy. On one hand, it has been reported that HCV core and NS5A proteins would generate LD aggregation (lipophagy inhibition), contributing to liver steatosis (fat accumulation) by a mechanism not fully understood (Mancone et al., 2011). Another study reported that the HCV core protein downregulates lipophagy in a model requiring DGAT1 for access to LD. Furthermore, the LD-localized core is consequently able to impair lipophagy, allowing LD accumulation and facilitating HCV assembly and steatosis (Harris et al., 2011). On the other hand, increased lipophagy was observed in HCV-infected HuH7 cells, suggesting a protective role for autophagy, as an inverse correlation between liver steatosis and lipophagy rates was found (Vescovo et al., 2012). Moreover, an induction of lipophagy during HCV infection is necessary and may contribute to the high ATP levels required for viral replication (Bose and Ray, 2014; Meyers et al., 2016).

Taken together, these findings allow hypothesizing the existence of an additional mechanism contributing to the release of HCV virions. This mechanism proposes the generation of extracellular LD (eLD) (positive for LC3) as a result of autophagy-mediated secretion. These eLD have been described to carry HCV infective RNA and HCV core and NS5A viral proteins. In fact, this mechanism may represent way to facilitate the spread of infectious HCV material from the host infected cell (Cloherty et al., 2020).

Several studies have confirmed that autophagy plays an important role in DENV/ZIKV infection; in fact, the blocking the autophagy pathway leads to a significant reduction in the viral replication rate (Heaton and Randall, 2010; Mateo et al., 2013; Jordan and Randall, 2017). In addition, an initial LD biogenesis upregulation has also been proposed, followed by an increase in lipophagy to drive virus production (Heaton and Randall, 2010; Cloherty et al., 2020). In this sense, a reduction in LD size was observed in DENV infected Huh-7.5 cells by electron microscopy (Heaton and Randall, 2010). Similar findings were reported in Huh-7 ZIKV infected cells in which a reduction in the LD numbers and total volume were observed, confirming an overall consumption of these organelles (Garcia et al., 2020). AUP1(ancient ubiquitous protein 1) is a multifunctional type III membrane protein that localizes predominantly to the ER and LD surface. AUP1 is involved in the LD accumulation and ER protein quality control, and has been proposed to act as a lipophagy-specific factor (Klemm et al., 2011). Specifically, DENV infection induces AUP1 deubiquitylation through a not fully understood mechanism. DENV NS4A and NS4B proteins bind and translocate AUP1 from the LD membrane to the LC3-decorated autophagosome surface, consequently upregulating lipophagy possibly through the AMPK/mTOR pathway (Randall, 2018; Wu et al., 2021). In this sense, the ZIKV proteins NS4A and NS4B have been found to bind and inhibit the Akt-mTOR pathway leading to lipophagy induction and defective neurogenesis in human neural stem cells (Liang et al., 2016). In addition, the knockout of AUP1 in HeLa and HepG2 cells leads to a decreased generation of infective DENV particles (Zhang et al., 2018).

It is suggested that eLD are also generated during DENV/ZIKV infection. In fact, it has been shown that cells infected by DENV/ZIKV were able to release LD inside secretory LC3-positive autophagosome structures, suggesting the existence of eLD originated from LD. Notably, DENV antigens, infective DENV RNA and LD have been found in secreted autophagosomes by Huh-7 infected cells (Wu et al., 2016). Besides, it has been hypothesized that for placental transmission. ZIKV would use a mechanism involving eLD through secretory autophagy (Zhang et al., 2016). Similarly, the bystander effect refers as a number of different not fully understood mechanisms allowing many viruses to establish intercellular communication in order to promote viral spreading (Palmer et al., 2005; Kofahi et al., 2016). In a recent study, an increase in the LD number and size in uninfected neighboring placental cells was found, suggesting a putative role for eLD bystander effect in the transmission of ZIKV infective components (Chen et al., 2020).

**FIGURE 4:**
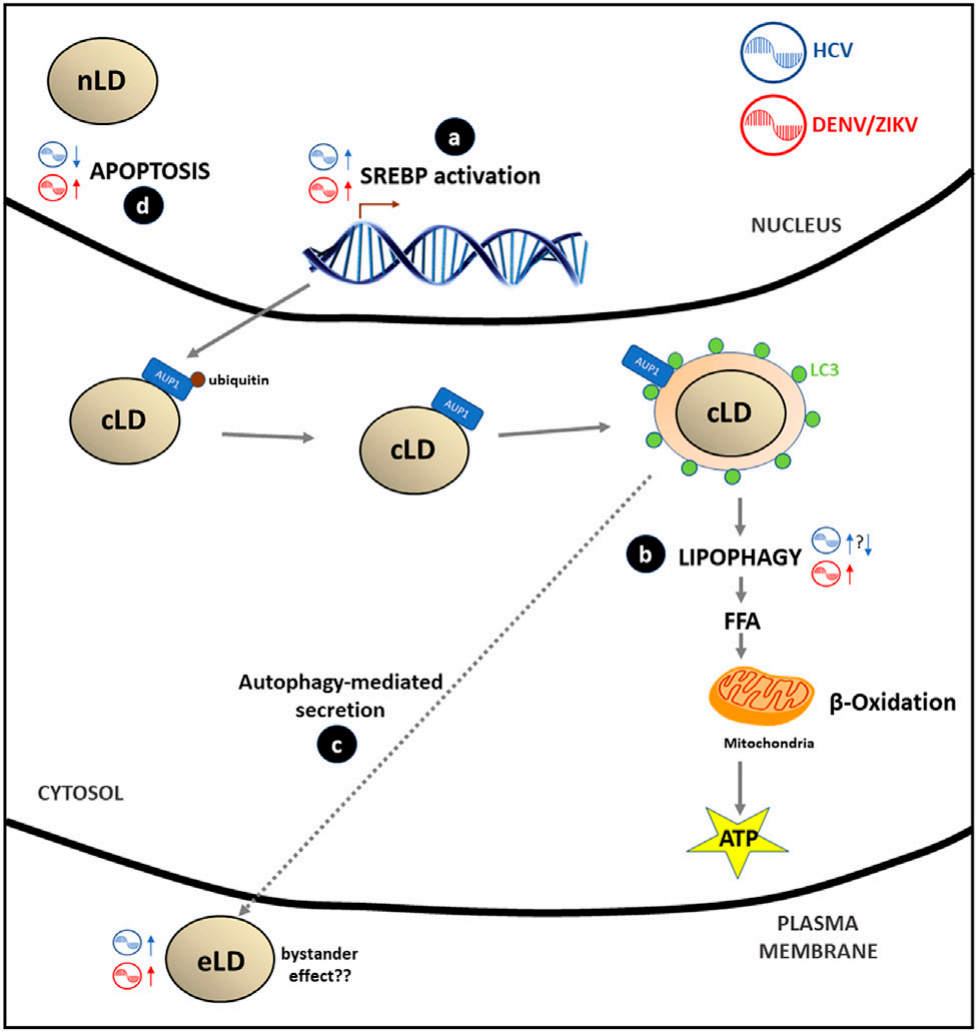
*Flaviviridae* effects in LD metabolism: **(A)** HCV, ZIKV/DENV stimulate the SREBP pathway through the transcription of genes involved in LD and lipids biosynthesis in order to cover the extra membrane requirement that virus replication generates. **(B)** DENV/ZIKV stimulates lipophagy by recruitment of deubiquitinated AUP1 from LD membrane to the LC3-positive autophagosome; this process generates FA that after catabolism inside the mitochondria (β-oxidation) produce energy (ATP) to accomplish viral replication. Data on the HCV effect on lipophagy are controversial: some authors report a stimulation of this process while other suggest that inhibition of lipophagy may occur. **(C)** Some authors hypothesize that there is a putative secretion of HCV or DENV/ZIKV virions, viral proteins or infectious viral RNA mediated by autophagy of LC3-positive LD vesicles (eLD) to spread the infection. This phenomenon may support the bystander effect proposed for DENV/ZIKV infections. **(D)** Apoptosis is inhibited (HCV) or stimulated (DENV/ZIKV) by viral and nLD interaction with PML nuclear bodies.

In summary, it is now clear that HCV and DENV/ZIKV take advantage of lipophagy as a source of energy for replication and for a possible extracellular spreading of the infective viral content through the generation of eLD. The detailed understanding of the molecular mechanism of lipophagy and its relationship with different *Flaviviridae* still needs further study and may set the scene for the development of novel antiviral treatments. All these evidences have been incorporated into Figure 4.

## Lipid Droplets in Cancer

In general, tumor cells are nutritionally challenged due to poor vascularization (Wellen and Thompson, 2010). Under deficient nutrient conditions, cells display a remarkable adaptability that is critical for survival, migration and invasion of other tissues (Pavlova and Thompson, 2016). In this context, tumor cells require energy suppliers to adapt to oxidative and nutritional stress conditions, allowing a rapid proliferation and progression of cancer. Lipids are an important energy reservoir that cancer cells can acquire from both exogenous and endogenous pools. Exogenous lipids are obtained from blood or from the tumoral microenvironment. On the other hand, the endogenous lipid availability depends on biosynthetic pathways, hydrolysis of membrane PL, autophagy, and LD (Petan et al., 2018). In addition to the capacity to obtain extracellular lipids, cancer cells have an efficient machinery to recycle intracellular lipids, which gives them a significantly higher probability of survival during hypoxia and starvation (Petan et al., 2018). Actually, some aggressive types of tumors have an increased capacity to accumulate FA in LD to resist nutrient and oxidative stress (Kamphorst et al., 2013; Padanad et al., 2016; Jarc et al., 2018; Kim et al., 2018). LD accumulation has been observed in many cancer cells such as colorectal, breast, prostate, hepatocellular carcinoma, renal carcinoma, and glioblastoma, suggesting that this organelle serves as a substrate for cell survival when the glucose levels decrease [recently reviewed in (Li et al., 2020)].

Several works have suggested that the accumulation of LD has a pro-tumoral role acting as sites of PGE2 synthesis (a suppressant of the immune system), in the polarization of tumor-associated macrophages in myeloid-derived cells, and on the dysfunctional antigen presentation by dendritic cells [recently reviewed in (Cruz et al., 2020)]. Recent studies suggest that these organelles suppress nutrient and oxidative stress and contribute to cancer cell survival and growth, metastasis, and resistance to chemotherapeutic and pharmacological treatments (Przybytkowski et al., 2007; Pucer et al., 2013; Bensaad et al., 2014; Welte and Gould, 2017; Cotte et al., 2018; Henne et al., 2018; Jarc et al., 2018; Pizato et al., 2019), suggesting that an in-depth study of LD metabolism could be an attractive target for reducing cancer cell resistance to stress. For this reason, LD accumulation in non-adipose tissues has been proposed as a new hallmark of cancer (Cotte et al., 2018).

### Lipid Droplet Biogenesis is Highly Regulated in Tumor Cells

Through tumorigenesis, cancer cells acquire different metabolic alterations to overcome the energetic requirement related to the accelerated proliferation under unfavorable conditions. Several studies have shown that some of these changes include the reprogramming of lipid metabolism such as *de novo* lipogenesis (Menendez and Lupu, 2007; Carracedo et al., 2013; Currie et al., 2013; Röhrig and Schulze, 2016). In contrast to normal cells, which preferentially use extracellular lipids for the synthesis of new structural lipids, cancer cells stimulate *de novo* FA synthesis to satisfy their requirements for lipids (Menendez and Lupu, 2007; Röhrig and Schulze, 2016). During *de novo* lipogenesis, saturated and monounsaturated FA are synthesized; nonetheless mammalian cells lack an enzyme capable of converting monounsaturated to polyunsaturated FA. This makes cancer cells more resistant to death from oxidative stress as well as drug therapy (Ameer et al., 2014). Even lipid-rich tumors have been associated with a high aggressive potential and an unfavorable clinical outcome (de Gonzalo-Calvo et al., 2015; Guillaumond et al., 2015).

As mentioned, SREBPs belong to a family of transcription factors bound to the ER membrane and, together with the mTOR, they act as key positive regulators of lipogenesis (Cruz et al., 2020). SREBPs have been shown to promote tumor growth as well as the accumulation of LD and the overexpression of the enzymes involved in lipogenesis. In addition, the SREBP cleavage-activating protein acts as a sensor for available glucose levels. It has been observed that the dysregulation of SREBPs occurs in several metabolic syndromes and cancers (Cheng et al., 2018). Moreover, SREBP as well as ATP Citrate Lyase (ACLY), a downstream target of SREBP, have been found to be upregulated in glioblastoma, colorectal cancer, breast cancer, non-small cell lung cancer, and hepatocellular carcinoma (de Gonzalo-Calvo et al., 2015; Guillaumond et al., 2015).

In mice with lung, prostate, or ovarian cancer xenografts, either the genetic or the pharmacological inhibition of SREBP and ACLY has been shown to significantly suppress tumor growth and induce cancer cell death (Hatzivassiliou et al., 2005; Hanai et al., 2013; Cheng et al., 2018), making SREBP and/or ACLY promising therapeutic targets (Infantino et al., 2007; Guo et al., 2009; Williams et al., 2013; Li et al., 2014; Geng et al., 2016). Interestingly, pre-clinical studies have demonstrated that some SREBP inhibitors such as fatostatin, botulin, and PF-429242 have promising anti-tumor effects (Kamisuki et al., 2009; Li et al., 2014, Li et al., 2015; Król et al., 2015; Gholkar et al., 2016; Shao et al., 2016). In addition, through the quantification of the mRNA expression levels, it has been demonstrated that LD coat proteins (PLIN) (Wang et al., 2018) and FA-binding proteins (FABP) are also involved in the regulation of LD formation and trafficking in cancer cells (Senga et al., 2018).

On the other hand, mTOR acts as a detector for the availability of extracellular nutrients, stimulating the activation of anabolic pathways such as protein translation and nucleotide synthesis. The PI3K/AKT/mTOR pathway regulates SREBP levels by promoting the synthesis of FA, cholesterol, and glycerolipids and is associated with an increase in the density of LD in tumor cells (Petan et al., 2018; Li et al., 2020).

Moreover, during starvation, the mTOR pathway is inhibited and the cell resorts to autophagy as a mechanism for the degradation of cytosolic components and membranous organelles to obtain FA available for LD biogenesis (Petan et al., 2018).

Under excess conditions, intracellular lipids are converted to TAG and SE in the ER, leading to the formation of LD (Fei et al., 2011; Walther and Farese, 2012). These structures have been visualized in several types of tumors including glioblastoma, renal clear cell carcinoma, and prostate, colon, or pancreas cancer (Accioly et al., 2008; Yue et al., 2014; Geng et al., 2016; Koizume and Miyagi, 2016). While in normal tissues SE are usually undetectable, they are abundant in the tumor tissue (Bemlih et al., 2010). Sterol *O*-acyltransferase 1 (SOAT1), also known as acyl-CoA acyltransferase 1 (ACAT1), converts cholesterol to SE for storage in LD. Interestingly, glioblastomas and prostate and pancreas cancers express high levels of this enzyme, being its expression level inversely correlated with patient survival (Bemlih et al., 2010; Saraon et al., 2014; Ohmoto et al., 2015; Geng et al., 2016, Geng et al., 2020; LaPensee et al., 2016; Li et al., 2016). The genetic silencing of SOAT1/ACAT1 or the pharmacologic blocking of its activity suppresses tumor growth in several cancer xenograft models (Bemlih et al., 2010; Ohmoto et al., 2015; Geng et al., 2016; LaPensee et al., 2016). These results suggest that SOAT1 and the synthesis of SE are two possible targets in the development of antitumor strategies.

Colorectal cancer (CRC) is one of the most common forms of cancer, in which the accumulation of LD appears to be a common feature (Tirinato et al., 2015; Kawasaki et al., 2017). The binding of the epidermal growth factor (EGF) to its receptor induces its activation, enabling downstream signaling pathways, including the PI3K/mTOR pathway, to induce cell proliferation and tumorigenesis, promoting the synthesis and accumulation of LD (Guri et al., 2017). Some authors postulate the existence of a negative regulatory loop between LD, the forkhead box transcription factor O-3 (FOXO3), and sirtuin 6 (a negative regulator of lipid biosynthesis) since the silencing of FOXO3 would promote the down-regulation of sirtuin 6 to increase LD levels (Penrose et al., 2016).

Breast cancer (BC) is the leading cause of cancer-associated death in women and the most common cancer worldwide (Bray et al., 2015). Several epidemiological studies have revealed that adipose tissue dysfunction appears to be one of the risk factors that contributes to the development and progression of BC. Given that aggressive BC cells have been shown to have a higher number of LD, and that obesity is a risk factor for breast cancer, some authors suggested an association between the alteration of LD homeostasis of the cancer cells and obesity (Wölwer et al., 2016; Blücher and Stadler, 2017).

Prostate cancer (PC) is the second leading cause of cancer-related death in men (Boettcher et al., 2019). PC cells can incorporate either circulating lipids or lipids from the adipose microenvironment to promote PC invasiveness through oxidative stress and the secretion of the hypoxia-inducible factor 1α (HIF-1α) (Diedrich et al., 2016; Victor, 2019). On the other hand, the *de novo* lipogenesis is also upregulated in PC cells and its inhibition suppresses PC growth both *in vitro* and *in vivo* (Yoshii et al., 2013). Cancer aggressiveness is positively correlated with LD density and LD movement speed during the transport of cargo proteins along microtubules (Yue et al., 2014). In addition, it has recently been described that autophagy and lipophagy are also associated with the aggressiveness and progression of PC, possibly through a mechanism that leads to the exploitation of a lipid-rich microenvironment by tumor cells (Panda et al., 2020).

Hepatocellular carcinoma (HCC) is the most common and aggressive liver cancer. One of the main pathological features of HCC is steatosis, which generally leads to an increase in the number of LD. PTEN (the phosphatase and tensin homologue on chromosome ten) has also been shown to be a negative regulator of the PI3K/AKT pathway and a classic tumor gene suppressor due to its lipid and protein phosphatase activity. The deletion of PTEN along with the overexpression of the *NRAS* proto-oncogene (RAS neuroblastoma) synergistically leads to a metabolic disorder that increases the LD content and promotes the appearance of HCC (Gao and Liu, 2017). Therefore, the accumulation of LD induced by the activation of oncogenic pathways could contribute to the development and progression of HCC. On the other hand, it is well known that SREBP1 plays a fundamental role in the progression of HCC as it promotes cancer cell growth and metastasis. It has recently been shown that Acyl-CoA Synthetase Long Chain 4 (ACSL4) enhances the expression of lipogenic enzymes through the *c-Myc/SREBP1* oncogene signaling; however more studies are needed to determine the association between ACSL4, metabolism and tumor lipid abnormalities (Chen et al., 2021).

Renal cell carcinoma (RCC) is one of the most common malignant tumors of the urinary system (Dutta et al., 2016). Among them, clear cell RCC (ccRCC) is the most common RCC subtype featured by an accumulation of LD. This carcinoma has a high risk of metastasis and a poor response to radiotherapy and chemotherapy (Gong et al., 2016). Patients with ccRCC display a high expression of PLIN3, and this phenomenon is closely correlated with clinicopathological features. Furthermore, the high expression of PLIN3 suggests a poor clinical prognosis (Wang et al., 2018). On the other hand, HIF2α promotes lipid storage, ER homeostasis, and cell viability in ccRCC through upregulation of the LD PLIN2 envelope protein. In conclusion, the study of the members of the perilipins family and the possible suppression of HIF2α/PLIN2 could be a useful tool for the development of therapeutic strategies in this common renal malignant neoplasm.

Glioblastoma (GBM) is a malignant tumor with lipid metabolism dysfunction (Guo et al., 2013; Cheng et al., 2015). Large amounts of LD are observed in tumor tissues of GBM patients that are not detectable in low-grade gliomas (Geng et al., 2016). Therefore, LD could be used as a diagnostic biomarker for GBM. When glucose supply decreases in GBM cells, LD are hydrolyzed by autophagy, thus explaining the survival of GBM cells in situations of energy stress (Geng et al., 2020). In addition, SREBP-1 has a high activity in GBM (Guo et al., 2011). The inhibition of SOAT1 down-regulates SREBP-1, resulting in a decrease in SE synthesis. Meanwhile, SOAT1 suppression reduces LD formation and consequently blocks GBM growth (Geng et al., 2016). Therefore, blocking the degradation of LD or the SREBP1/SOAT1 pathway would be a suitable therapeutic strategy to increase the sensitivity of GBM to treatments and overcome resistance.

To summarize, the accumulation of LD in cancer cells depends on the activation of SREBP and mTOR pathways, suggesting that both pathways are important in cancer development and progression.

### Lipolysis and Lipophagy in Cancer

**FIGURE 5:**
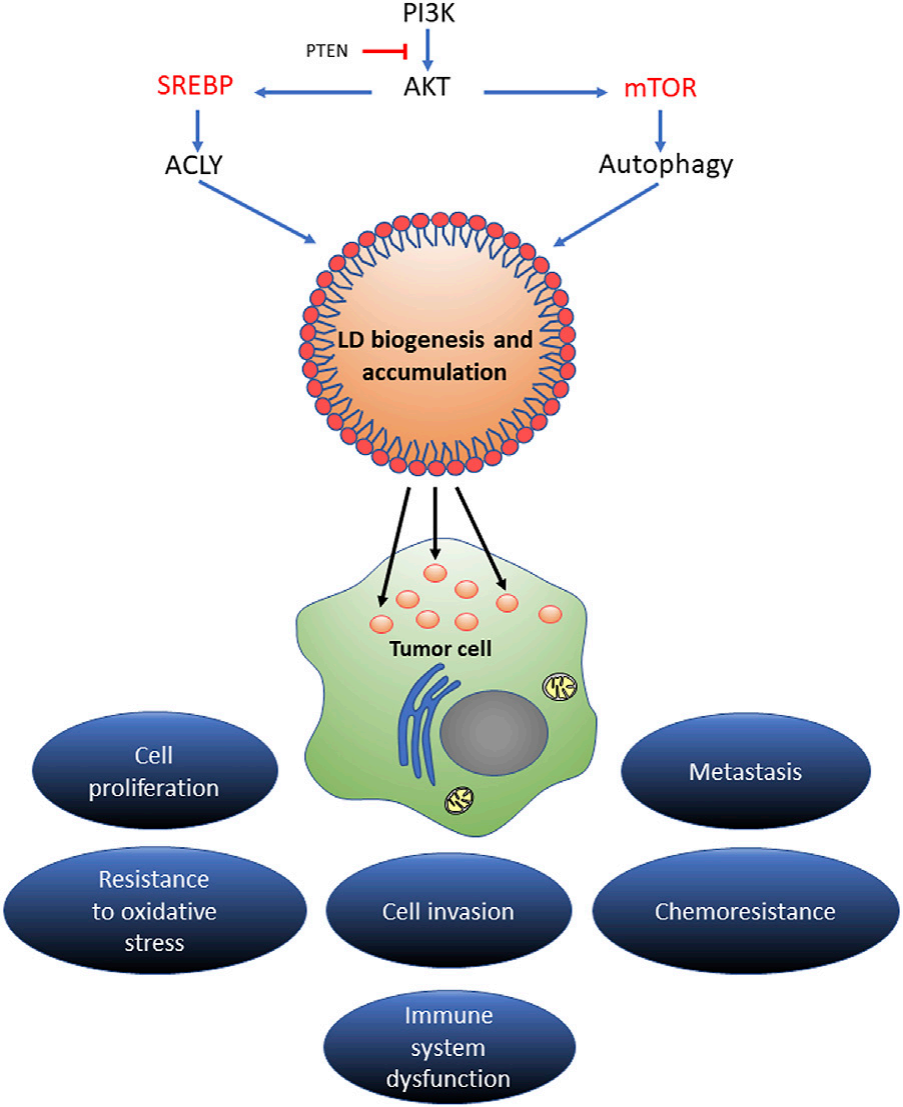
The regulatory mechanism of LD in cancer progression. Different signaling pathways of lipid acquisition may drive LD biogenesis in stressed cancer cells. Increased LD contents could expand the source of lipid substrates and energy to meet the metabolic needs of proliferating cancer cells. In the tumor microenvironment, LD could act as an energy reservoir for an aggressive cancer to trigger metastatic cloning. LD accumulation extensively mediates proliferation, invasion, metastasis, and oxidative stress and chemotherapy resistance in multiple types of cancers.

As mentioned above, the energy demand can drive the degradation of accumulated LD in the cell, mainly by two mechanisms, lipolysis or lipophagy. Although several authors have shown that lipophagy has pro-tumoral effects (Kaini et al., 2012; Assumpção et al., 2017), most studies performed so far suggest that lipophagy restricts tumorigenesis (Xu et al., 2016; Mukhopadhyay et al., 2017). Moreover, it has been shown that the overexpression of ATG14, a member of the ATG proteins, induces LD breakdown in Hela cells (a cervical cancer cell line) and stimulates free FA accumulation. This process leads to ER stress and reactive oxygen species-mediated apoptosis, whereas the inhibition of lipophagy or the inhibition of lysosomal acid lipases (LAL) reverts these effects (Mukhopadhyay et al., 2017). LAL plays a tumor suppressor role and its deficiency in mice has been linked with spontaneous tumorigenesis. In contrast, the re-expression of LAL prevents liver metastases (Du et al., 2015) and reduces inflammation and metastasis in lung cancer (Zhao et al., 2016). On the other hand, it has been shown that when autophagy is inhibited in adipocytes, LD clearance is slowed down and consequently the effects that promote adipocyte growth are attenuated. In other words, in this cell type, lipophagy is activated in order to promote the production of energy and the survival of cancer cells (Petan et al., 2018). It has been demonstrated that abhydrolase domain-containing protein 5, a cellular lipolytic activator, which functions as a tumor suppressor in CRC, binds and prevents the cleavage of the essential autophagy regulator beclin 1, thus stimulating autophagy, reducing colon cancer tumorigenesis (Peng et al., 2016). Additionally, it has been demonstrated that ATGL promotes the autophagic flux and the interactions between LC3 and LD (Drizyte-Miller et al., 2020). In turn, LC3 depletion results in reduced LD accumulation in various cancer cell lines such as HeLa, HepG2, and PC12 (Shibata et al., 2010). Together, these results suggest an important role for LC3 in the formation of LD. Interestingly, rapamycin-induced autophagy leads to TAG synthesis in yeast (Madeira et al., 2015), and autophagy is necessary for TAG accumulation under nitrogen-deprived conditions in this microorganism. In conclusion, autophagy-driven LD synthesis is helpful for the progression of a variety of cancer cells. Further studies should confirm whether LD biogenesis is a protective response to high levels of autophagy. All these evidences have been incorporated into Figure 5.

## Concluding Remarks

As mentioned, the biology of LD has recently received the attention of multiple research fields, opening a wide window of knowledge in science. Given that LD are conserved structures in prokaryotic and eukaryotic cells (Walther et al., 2017; Zhang and Liu, 2017; Lupette and Maréchal, 2020), a role in every studied biological process should not be surprising. Pathogen invasion, as well as the unlimited proliferation of cancer cells, are processes that impose a huge cellular remodeling, with a consequent energy demand satisfied by LD metabolism.

In general, studies describing LD changes during infections and cancer focus on metabolic aspects and organelle patterns at the cellular level (LD size and number) associated with different disease stages. The bridge between these approaches is currently being disentangled as considerable advances have been done in the study of the LD biogenesis and degradation processes. Some of these metabolic aspects are: 1) the increase in TAG levels, which leads to an increase of DGAT activity (parasitic protozoans), *de novo* FA synthesis, (e.g., SREBP upregulation in HCV and DENV/ZIKV replication and cancer cells), and increase in FA uptake (cancer cells), and 2) the free cholesterol/SE balance, which is modulated by SREBP (cancer) or aspartyl-like peptidase (e.g., *T. cruzi*) and the enzyme ACAT, which might display a key role in the parasite’s survival (e.g., *T. gondii*).

In cancer cells, *de novo* lipogenesis enhancement leads to the generation of cell membranes that are enriched in saturated and/or mono-unsaturated FA (as polyunsaturated FAs cannot be synthesized by this pathway) (Ameer et al., 2014). In turn, NL saturation has been observed to respond to demands of unsaturated species in the cell (providing a homeostasis mechanism of membrane saturation by “buffering” specific FA). For instance, the TAG saturation index increases as a consequence of the liberation of unsaturated FA to counteract their esterification and transformation into phospholipids when cancer cells are subjected to nutrient stress (Lisec et al., 2019) or inhibition of the *de novo* desaturation pathway through hypoxia (Ackerman et al., 2018).

Tumor tissues contain abnormal levels of SE (Nygren et al., 2009; Bemlih et al., 2010). In *T. cruzi*, the cholesterol content variation leads to morphological changes (on both reservosomes and cytosolic LD) arising from NL crystallization in the core of these structures. This suggests that significant biophysical differences between the stages of LD biogenesis as well as on protein targeting to LD surface could be taking place. The latter findings deserve further exploration. The requirement of sterols for the coordinated assembly of LD seems to be universal. Recently, this aspect has been studied in *Arabidopsis* developing seeds to find that the mutations of proteins of the sterol pathway account for the different LD number, size and oil content phenotypes (Yu et al., 2021) and some interesting comparisons with cholesterol effects arise. For instance, cholesterol generates lipid packing defects and increases the surface tension of membranes synergistically with DAG (Coorssen and Rand, 2011; Alwarawrah et al., 2012; Subczynski et al., 2017), which has been suggested to be critical for neutral lipid nucleation and LD budding (Adeyo et al., 2011; Choudhary et al., 2018). In this sense, in a study that combined molecular simulations, yeast genetics, and fluorescence microscopy (Zoni et al., 2021), it has been recently demonstrated that cholesterol promotes LD nucleation and the packaging of TG into LD.

Taking into account the above considerations, current experimental models using biophysical approaches may help address the following questions that arise from metabolic observations: how NL composition (cholesterol:cholesterol-esters:triglyceride proportions) affect the biogenesis process? How do properties (unsaturation and carbon length) of FA constituting neutral lipids affect biogenesis?

Regarding protein targeting to LD, a putative effect of the LD core composition has also been considered. The interaction of amphipatic helices (AH) to LD model surface was sensitive to the core composition (Dhiman et al., 2020). Surprisingly, this effect was independent of the phospholipid monolayer packing. On the other hand, cholesterol has been demonstrated to affect the physical properties of the LD surface and hence the targeting of TG-synthesizing enzymes to LD (Wilfling et al., 2014). In this sense, PLIN4 has served as an interesting model protein due to its exceptional length and repetitiveness that confers it versatility to compensate between those properties (AH length, hydrophobicity, and charge) targeting it to the LD surface, although with a loss of specificity (Čopič et al., 2018). How changes in the NL composition of LD, promoted in infections and cancer, can impact on their interaction with AH containing proteins remains to be assessed.

Finally, LD degradation helps cells coping with the high energy demands in pathological processes; therefore, this process could serve as a target for the development of novel therapeutic approaches.
